# Plasma mitochondrial DNA levels are associated with acute lung injury and mortality in septic patients

**DOI:** 10.1186/s12890-021-01437-2

**Published:** 2021-02-25

**Authors:** Jia-yu Mao, Dong-kai Li, Hong-min Zhang, Xiao-ting Wang, Da-wei Liu

**Affiliations:** Department of Critical Care Medicine, Peking Union Medical College Hospital, Peking Union Medical College and Chinese Academy of Medical Science, 1 Shuaifuyuan Road, Dongcheng District, Beijing, 100730 China

**Keywords:** Plasma mtDNA level, Biomarkers, Acute lung injury, Sepsis

## Abstract

**Background:**

Mitochondrial DNA (mtDNA) is a critical activator of inflammation. Circulating mtDNA released causes lung injury in experimental models. We hypothesized that elevated plasma mtDNA levels are associated with acute lung injury (ALI) in septic patients.

**Methods:**

We enrolled 66 patients with sepsis admitted to the Department of Critical Care Medicine of Peking Union Medical College Hospital between January 2019 and October 2019. Respiratory, hemodynamic and bedside echocardiographic parameters were recorded. Plasma mtDNA, procalcitonin, interleukin 6, and interleukin 8 levels were examined.

**Results:**

Plasma mtDNA levels within 24 h after admission were significantly increased in the group of septic patients with ALI [5.01 (3.38–6.64) vs 4.13 (3.20–5.07) log copies/µL, p 0.0172]. mtDNA levels were independently associated with mortality (hazard ratio, 3.2052; 95% CI 1.1608–8.8500; p 0.0253) and ALI risk (odds ratio 2.7506; 95% CI 1.1647–6.4959; p 0.0210). Patients with high mtDNA levels had worse outcomes, and post hoc tests showed significant differences in 28-day survival rates. Increased mtDNA levels were seen in patients with abdominal infection.

**Conclusions:**

Increased plasma mtDNA levels within 24 h after admission were significantly associated with ALI incidence and mortality in septic patients.

## Background

Mitochondrial DNA (mtDNA), as a potential damage-associated molecular pattern (DAMP), has attracted intense research in recent years since it may contribute to the mechanism by which mitochondria regulate innate immunity [1]. Cell stress and necrosis caused by injury may result in fragmentation of the mitochondrial genome and disruption of the mitochondrial membrane, ultimately leading to extracellular release of mitochondrial DNA [2, 3]. Circulating mtDNA is capable of triggering innate immunity through multiple mechanisms, such as activating the Toll-like receptor 9 (TLR-9)/NF-κB pathway or NALP3 inflammasome [4, 5]. As a consequence, mtDNA may amplify the injury and contribute to the persistent and dysregulated inflammatory response.

Growing evidence has revealed that extracellular mtDNA might act as a biomarker in critically ill patients. Elevated levels of plasma mtDNA are associated with sepsis, trauma or postcardiac arrest [1, 6–9]. Intervention with mtDNA in an animal study induced systemic inflammatory response syndrome (SIRS) and pulmonary edema [4], and blockade of TLR9 improved the lung histopathological changes, wet/dry ratios and inflammatory factor concentrations [10]. mtDNA has been shown to increase endothelial cell permeability, either directly or through interactions with endothelial cells and polymorphonuclear leukocytes [11]. Therefore, circulating mtDNA may account for lung injury during nonpulmonary insults, such as sepsis.

In this study, we explored the relationship between circulating mtDNA and septic lung injury, especially at extrapulmonary infection sites. We hypothesized that plasma mtDNA levels may reflect the level of septic organ dysfunction and prognosis.

## Methods

### Study design

We performed a prospective study among patients in the Department of Critical Care Medicine of Peking Union Medical College Hospital (PUMCH) from January 2019 to October 2019, which was approved by the PUMCH institutional review board (approval number JS-1985). All methods were performed in accordance with the relevant guidelines and regulations, informed consent was obtained from all enrolled patients through the next of kin of each patient. The inclusion criteria in this study were as follows: (1) age ≥ 18 years, (2) intensive care unit (ICU) stay of more than 24 h, and (3) sepsis diagnosis (see below). Patients who were younger than 18 years old, discharged within 24 h after admitted, or had basic pulmonary disease were excluded.

Sepsis was defined according to consensus international guidelines as life-threatening organ dysfunction caused by a dysregulated host response to infection [12]. Organ dysfunction was defined as an acute change in total Sequential Organ Failure Assessment (SOFA) score of ≥ 2 points consequent to the infection. Acute lung injury (ALI) was considered to be present if PaO_2_/FiO_2_ (oxygen index, OI) ≤ 300 mmHg [13]. The enrolled patients were divided into a control group composed of patients without ALI and an ALI group. The sample size was estimated based on a priori power calculation indicating an 80% power to detect a difference of mtDNA copy number frequency (effect size 0.7) between control and ALI group at the 0.05 significance level using a power and sample size website (http://powerandsamplesize.com).

### Outcome

Vital sign tests, laboratory tests, echocardiography, analysis of plasma mtDNA copies and clinical history evaluation were carried out on the first day after ICU admission. Basic clinical and laboratory characteristics included age, sex, basic cardiological or pulmonary disease, hemodynamic parameters, respiratory parameters, blood chemistry, arterial blood gas analysis, Acute Physiology and Chronic Health Evaluation (APACHE) II score, and SOFA score. Life-sustaining treatment included mechanical ventilation, vasopressor administration, and renal replacement. Follow-up data included ICU stay time and 28-day mortality rate.

Peripheral blood samples were collected within 24 h of ICU admission and centrifuged immediately, and the plasma was withdrawn and stored at − 80 °C until assessment of mtDNA copies. Plasma mtDNA was extracted using a commercially available kit (DP 318, Tiangen Biochemistry, Beijing), quantified using polymerase chain reaction (PCR) for the mitochondrial ND1 gene and measured in triplicate as reported previously. The fold‑change was calculated by the 2^‑ΔΔCq^ method compared with standards in the kit and showed using log-transformed copy number/µL to ensure normality [6, 14]. The primers for the mtND1 gene were forward 5′-ATACCCATGGCCAACCTCCT-3′ and reverse 5′-GGGCCTTTGCGTAGTTGTAT-3′.

New onset RV dysfunction was assessed using multimodality parameters as defined by the American Society of Echocardiography (ASE) criteria, i.e., specifically semiquantitative size and function, tricuspid annular plane systolic excursion (TAPSE) < 16 mm by M-mode, tricuspid lateral annulus tissue Doppler systolic velocity < 0.15 cm/s or RV fractional area change < 35% [15]. Echocardiographic examinations were performed by two experienced intensivists. An ultrasonic machine (M-Turbo Sonosite, Highland Heights, Ohio) equipped with a 15 MHz transducer was used for noninvasive transthoracic echocardiography. Data from 3 consecutive cardiac cycles were analyzed and averaged.

### Statistical analysis

The normality of the data was evaluated using the Kolmogorov–Smirnov test. Normally distributed data were expressed as the mean and standard deviation and were compared using Student’s t-test. Nonnormally distributed data are presented as the median, and interquartile intervals were analyzed with the Mann–Whitney U test. Categorical variables were recorded as proportions. Univariate logistic regression was performed to identify mtDNA and other parameters with predictive value for 28-day mortality and ALI risk. The Cox proportional hazard survival model was applied to identify the independent contribution of prognostic factors to the prediction of the 28-day outcome. The results are expressed as the Wald index, odds ratio (OR), hazard ratio (HR) and 95% CIs. Discrimination of values was calculated using receiver operating characteristic (ROC) curve analysis using the Hanley-McNeil test. Survival curves up to day 28 were estimated using the Kaplan–Meier method, and the log-rank (Mantel–Cox) test was used to estimate differences among the predefined groups. All comparisons were two-tailed, and a p value less than 0.05 was required to exclude the null hypothesis. Statistical analyses were performed using the SPSS 13.0 software package (SPSS, Chicago, IL).

## Results

### Basic characteristics

During the study period (from January to October 2019), 101 patients with sepsis were admitted to the Critical Care Medicine Department of PUMCH. Of these, 32 satisfied at least 1 of the exclusion criteria (3 were < 18 years old, 21 were discharged with 24 h after admission, and 8 had basic pulmonary disease), and 3 patients refused to sign the consent form. A total of 66 patients were enrolled in the study, 40 of whom met the ALI criteria.

**Fig. 1:**
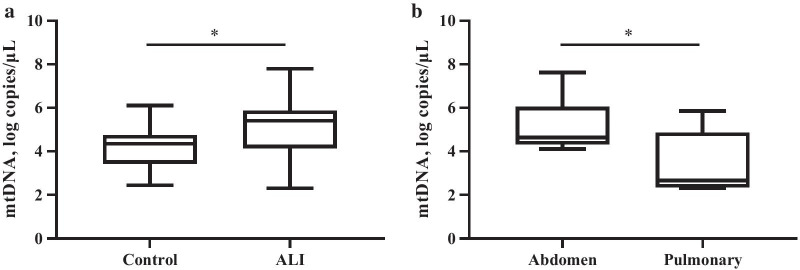
Comparison of mtDNA level in different groups. **a** MtDNA level in septic patients with or without acute lung injury. **b** MtDNA level in septic ALI patients with different infectious sources. *ALI* acute lung injury. *p < 0.05

The basic clinical characteristics of all patients included in this study after ICU admission are shown in Table 1. The majority of patients were male, and elderly individuals accounted for a large portion of the ALI patients. The composition of pulmonary infection sources was accordant in patients with or without lung injury; however, bloodstream and abdominal infections seemed to be more common in patients with ALI. The mortality, ICU stay time and APACHE II and SOFA scores were significantly higher in the ALI group than in the control group. In terms of respiratory condition, the OI, plat pressure (Pplat), and positive end expiratory pressure (PEEP) were worse in the ALI group. Regarding hemodynamic data, compared with those in the control group, patients with ALI had a higher central venous pressure (CVP), and no significant difference in mean arterial pressure (MAP), heart rate (HR) or norepinephrine (NE) dose was seen. Only mtDNA [5.01 (3.38–6.64) vs 4.13 (3.20–5.07), p 0.0172] (Fig. 1a) as a biomarker rather than procalcitonin (PCT), C-reactive protein (CRP) or other inflammatory factors, such as interleukin 6 (IL-6), interleukin 8 (IL-8), interleukin 10 (IL-10) or tumor necrosis factor α (TNFα), showed significant differences between the different groups.

**Table 1:** The general characteristics of the patients included in this study

Characteristics	Control, n = 26	ALI, n = 40	p
Age, years	49.7 (31.8–67.7)	65.6 (51.7–79.4)	0.0002***
Sex, n (%)			
Male	14 (53.8)	28 (70.0)	
Infection site, n (%)			
Blood	3 (11.5)	11 (27.5)	
Lung	10 (38.5)	14 (35.0)	
Abdomen	4 (15.4)	10 (25.0)	
Other	9 (34.6)	5 (12.5)	
Mortality, n (%)	0 (0.0)	12 (30.0)	
Ventilator-free time, days	3.1 (0.3–5.9)	17.9 (1.9–34.0)	< 0.0001***
ICU stay time, days	4.5 (3.0–6.8)	15.5 (8.5–33.5)	< 0.0001***
APACHE II	15.7 (7.7–23.6)	22.5 (12.0–33.0)	0.0069**
SOFA	6.0 (3.4–8.6)	9.5 (6.0–13.1)	< 0.0001***
OI, mmHg	374 (329–420)	220 (170–270)	< 0.0001***
PEEP, cmH2O	5.2 (4.7–5.7)	7.0 (5.0–9.0)	< 0.0001***
Pplat, cmH2O	18.0 (15.2–20.9)	20.5 (16.0–25.0)	0.0226*
MAP, mmHg	81.3 (74.3–88.2)	84.3 (74.5–94.1)	0.1835
HR, bpm	94.2 (78.2–110.1)	94.9 (74.2–115.5)	0.8861
CVP, mmHg	8.4 (6.9–10.0)	9.8 (7.9–11.8)	0.0063**
NE, µg/kg/min	0.19 (0.02–0.29)	0.18 (0.08–0.38)	0.0986
Lactate, mmol/L	2.1 (0.8–3.4)	2.2 (0.3–4.2)	0.7388
mtDNA, log copies/µL	4.13 (3.20–5.07)	5.01 (3.38–6.64)	0.0172*
PCT, ng/mL	7.0 (3.8–19.0)	5.9 (2.0–20.3)	0.4274
CRP, mg/L	127 (79–175)	125 (72–177)	0.8537
IL-6, pg/mL	139 (49–376)	93 (28–145)	0.8147
IL-8, pg/mL	157 (42–274)	131 (53–247)	0.1971
IL-10, pg/mL	12 (6–21)	8 (5–14)	0.2418
TNFα, pg/mL	13 (9–19)	21 (13–30)	0.8210

*ALI* acute lung injury, *ICU* intensive care unit, *APACHE* Acute Physiology and Chronic Health Evaluation, *SOFA* Sequential Organ Failure Assessment, *OI* oxygen index, *PEEP* positive end expiratory pressure, *Pplat* plat pressure, *MAP* mean arterial pressure, *HR* heart rate, *CVP* central venous pressure, *NE* norepinephrine, *PCT* procalcitonin, *CRP* C-reactin protein, *IL-6* interleukin 6, *IL-8* interleukin 8, *IL-10* interleukin 10, *TNFα* tumor necrosis factor α

### Risk factors for mortality in sepsis patients

A univariate logistic regression analysis indicated that variables including mtDNA level, APACHE II score, SOFA score, OI, PEEP, CVP and NE level were associated with mortality in septic patients. Cox proportional hazard survival model was used then to adjust relevant variables to examine possible risk factors for a poor prognosis. The HR of mtDNA after adjusted was 3.2052 (95% CI 1.1608–8.8500; p 0.0253) (Tables 2, 3).

**Table 2:** Univariate logistic regression analysis for possible risk factors for prognosis

Variable	B	SE	Wald	p	OR	95% CI for OR	
						Lower	Upper
Age	0.0408	0.0224	3.9240	0.0681	1.0417	0.9970	1.0884
APACHE II	0.0975	0.0339	9.9070	0.0040**	1.1024	1.0315	1.1780
SOFA	0.2714	0.1039	8.2880	0.0090**	1.3118	1.0701	1.6080
mtDNA	1.4154	0.4259	21.5720	0.0009***	4.1182	1.7872	9.4891
OI	− 0.0148	0.0054	10.8110	0.0063**	0.9853	0.9750	0.9958
PEEP	0.5187	0.1807	9.6210	0.0041**	1.6799	1.1789	2.3936
Pplat	0.1385	0.0749	3.4870	0.0642	1.1486	0.9918	1.3301
CVP	0.3689	0.1819	4.4290	0.0425*	1.4461	1.0125	2.0653
NE	3.7879	1.1700	14.8870	0.0012**	44.1655	4.4579	437.5610
PCT	0.0184	0.0099	3.243	0.0643	1.0185	0.9989	1.0386

**Table 3:** Cox regression analysis for possible risk factors for prognosis

Variable	B	SE	p	HR	95% CI for OR	
					Lower	Upper
SOFA	0.3348	0.1675	0.0457*	1.3976	1.0081	1.9377
mtDNA	1.1648	0.5208	0.0253*	3.2052	1.1608	8.8500
OI	0.0116	0.0077	0.1343	1.0116	0.9965	1.0270
PEEP	− 0.1750	0.2217	0.4300	0.8395	0.5448	1.2935
CVP	− 0.7199	0.3540	0.0420*	0.4868	0.2441	0.9709
NE	1.7602	0.8862	0.0470*	5.8138	1.0326	32.7319

*APACHE* Acute Physiology and Chronic Health Evaluation, *SOFA* Sequential Organ Failure Assessment, *OI* oxygen index, *PEEP* positive end expiratory pressure, *CVP* central venous pressure, *NE* norepinephrine

### The mtDNA level was associated with mortality

**Fig. 2:**
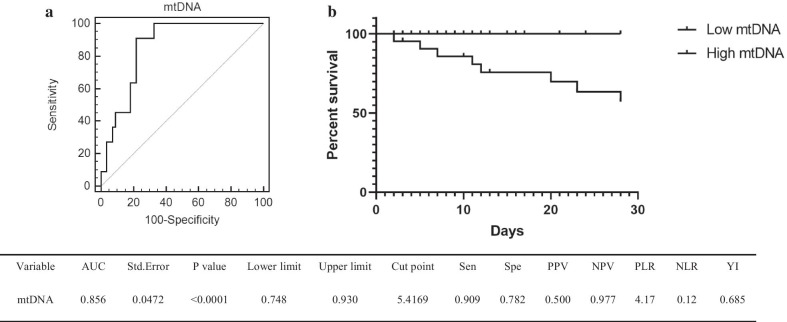
The relationships between mtDNA level and the prognostic outcome. **a** The cutoff value of mtDNA was calculated from the receiver operating characteristic curve, and this value was used to determine the prognostic significance. **b** The cutoff values of mtDNA level were used to determine the prognostic significance. *AUC* area under the curve, *NLR* negative likelihood ratio, *PLR* positive likelihood ratio, *PPV* positive predictive value, *YI* Youden index

The ROC curve was drawn for mortality (Fig. 2a). The area under the curve for mtDNA was 0.856 (95% CI 0.748–0.930, p < 0.0001). The cutoff value for mtDNA was equal to 5.4169 log copies/µL, based on the maximum Youden index. Based on the mtDNA cutoff, all the patients were divided into a low mtDNA group and a high mtDNA group. Post hoc tests showed significant differences in 28-day survival rates between the different groups [log-rank (Mantel–Cox), 6.842; p 0.0089] (Fig. 2b).

The clinical characteristics of patients with different mtDNA amounts are shown in Table 4. There were no significant differences in age, sex, or underlying cardiac disease between the different groups. The mortality (50% vs 2.3%), ICU stay time [21.5 (11.3–42.8) vs 6.0 (4.0–12.0) days, p < 0.0001], APACHE II score [29.0 (20.0–38.0) vs 15.2 (8.1–22.3), p < 0.0001] and SOFA score [10.9 (7.6–14.2) vs 6.8 (3.8–9.7), p < 0.0001] were significantly higher in the group of patients with high mtDNA than in the patients with low mtDNA. Both respiratory and hemodynamic conditions worsened in the high mtDNA group. In terms of respiratory condition, the OI [222 (156–289) vs 339 (255–422) mmHg, p 0.0002], PEEP [7.4 (5.2–9.7) vs 5.8 (4.6–7.0) cmH2O, p 0.0005], and Pplat [21.5 (16.8–26.1) vs 18.6 (15.2–22.0) cmH2O, p 0.0080] were significantly worse in the high mtDNA group. Regarding hemodynamic data, compared with the control group patients, patients with high mtDNA had a higher lactate value [3.0 (0.6–5.4) vs 1.8 (0.7–2.8) mmol/L, p 0.0051], CVP [10.4 (8.8–12.0) vs 8.4 (6.9–9.9) mmHg, p 0.0005], NE dose [0.27 (0.15–0.87) vs 0.17 (0–0.28) µg/kg/min, p 0.0006], and RV dysfunction occurrence (59.1% vs 15.9%). Levels of other inflammatory biomarkers, such as PCT [7.6 (2.5–52.5) vs 6.0 (2.3–19.0) ng/mL, p 0.0091], IL-6 [130 (48–966) vs 84 (36–271) pg/mL, p 0.0280], and IL-8 [186 (58–458) vs 131 (42–222) pg/mL, p 0.0121], showed significant differences in patients with different mtDNA amounts.

**Table 4:** Laboratory data parameters in the different groups

Characteristics	Low mtDNA, n = 44	High mtDNA, n = 22	p
Age, years	57.1 (39.3–88.3)	63.8 (48.5–79.2)	0.1436
Sex, n (%)			
Male	28 (63.6)	13 (59.1)	
Infection site			
Blood	2 (4.5)	12 (54.5)	
Lung	20 (45.5)	4 (18.2)	
Abdomen	9 (20.5)	5 (22.7)	
Other	13 (29.5)	1 (4.6)	
Mortality, n (%)	1 (2.3)	11 (50)	
Ventilator-free time, days	6.0 (1.8–6.7)	22.3 (8.0–33.0)	< 0.0001***
ICU stay time, days	6.0 (4.0–12.0)	21.5 (11.3–42.8)	< 0.0001***
RV dysfunction	7 (15.9)	13 (59.1)	
APACHE II	15.2 (8.1–22.3)	29.0 (20.0–38.0)	< 0.0001***
SOFA	6.8 (3.8–9.7)	10.9 (7.6–14.2)	< 0.0001***
OI, mmHg	339 (255–422)	222 (156–289)	0.0002***
PEEP, cmH2O	5.8 (4.6–7.0)	7.4 (5.2–9.7)	0.0005***
Pplat, cmH2O	18.6 (15.2–22.0)	21.5 (16.8–26.1)	0.0080**
MAP, mmHg	83.4 (75.3–91.6)	82.4 (72.2–92.7)	0.6809
HR, bpm	92.0 (76.2–107.8)	99.7 (76.5–122.9)	0.1216
CVP, mmHg	8.4 (6.9–9.9)	10.4 (8.8–12.0)	0.0005***
NE, µg/kg/min	0.17 (0–0.28)	0.27 (0.15–0.87)	0.0006***
Lactate, mmol/L	1.8 (0.7–2.8)	3.0 (0.6–5.4)	0.0051**
PCT, ng/mL	6.0 (2.3–19.0)	7.6 (2.5–52.5)	0.0091**
IL-6, pg/mL	84 (36–271)	130 (48–966)	0.0280*
IL-8, pg/mL	131 (42–222)	186 (58–458)	0.0121*
IL-10, pg/mL	8 (5–15)	12 (6–47)	0.2038
TNFα, pg/mL	15 (9–22)	26 (18–36)	0.5511

*ICU* intensive care unit, *RV* right ventricular, *APACHE* Acute Physiology and Chronic Health Evaluation, *SOFA* Sequential Organ Failure Assessment, *OI* oxygen index, *PEEP* positive end expiratory pressure, *Pplat* plat pressure, *MAP* mean arterial pressure, *HR* heart rate, *CVP* central venous pressure, *NE* norepinephrine, *PCT* procalcitonin, *CRP* C-reactin protein, *IL-6* interleukin 6, *IL-8* interleukin 8, *IL-10* interleukin 10, *TNFα* tumor necrosis factor α

### mtDNA was associated with ALI risk in sepsis patients

A univariate logistic regression analysis was used to examine ALI risk in sepsis patients. The variables mtDNA, age, APACHE II score, SOFA score, Pplat and PEEP were indicated to be associated with ALI risk in septic patients. A multivariate logistic regression analysis indicated that, the OR of mtDNA after adjusted was 2.7506 (95% CI 1.1647–6.4959, p 0.0210) (Tables 5, 6).

**Table 5:** Univariate logistic regression analysis for possible risk factors for ALI

Variable	B	SE	p	OR	95% CI for OR	
					Lower	Upper
Age	0.0461	0.0171	0.0070**	1.0472	1.0127	1.0828
APACHE II	0.1848	0.0517	0.0003***	1.2030	1.0871	1.3313
SOFA	0.4558	0.1203	0.0002***	1.5774	1.2461	1.9970
mtDNA	0.9020	0.2457	0.0002***	2.4644	1.5226	3.9889
PEEP	0.7010	0.2578	0.0065**	2.0158	1.2161	3.3411
Pplat	0.1629	0.0787	0.0385*	1.1769	1.0086	1.3732

*APACHE* Acute Physiology and Chronic Health Evaluation, *SOFA* Sequential Organ Failure Assessment, *OI* oxygen index, *PEEP* positive end expiratory pressure, *CVP* central venous pressure, *NE* norepinephrine

**Table 6:** Multivariate logistic regression analysis for possible risk factors for ALI

Variable	B	SE	p	OR	95% CI for OR	
					Lower	Upper
Age	0.0212	0.0247	0.3904	1.0215	0.9731	1.0722
APACHE II	0.0541	0.0604	0.3708	1.0555	0.9377	1.1882
SOFA	0.3630	0.1791	0.0427*	1.4376	1.0120	2.0422
mtDNA	1.0118	0.4385	0.0210*	2.7506	1.1647	6.4959
PEEP	0.3742	0.3799	0.3247	1.4539	0.6904	3.0615
Pplat	0.0804	0.1162	0.4887	1.0838	0.8631	1.3609

*APACHE* Acute Physiology and Chronic Health Evaluation, *SOFA* Sequential Organ Failure Assessment, *OI* oxygen index, *PEEP* positive end expiratory pressure, *CVP* central venous pressure, *NE* norepinephrine

### Comparison of mtDNA in ALI patients with different infectious sources

Patients with different infectious sources also showed different clinical characteristics (Table 7). No difference was shown in age, sex, ICU stay time, APACHE II and SOFA scores between patients with abdominal or pulmonary infection. However, the OI of abdominal infection patients was even worse than that of pulmonary infection patients [215 (178–243) vs 256 (230–271) mmHg, p 0.0369]. Patients with abdominal infection showed higher HR [100.0 (95.0–104.5) vs 84.0 (70.0–91.0) bpm, p 0.0176] and NE dose [0.23 (0.06–0.73) vs 0.11 (0.05–0.18) µg/kg/min, p 0.0427] than patients with pulmonary infection. Higher amounts of mtDNA (Fig. 1b) and other inflammatory biomarkers were seen in patients with abdominal infection [mtDNA, 5.14 (4.39–5.87) vs 4.79 (2.52–5.54) log copies/µL, p 0.0492; PCT, 8.1 (3.2–93.3) vs 2.0 (0.8–6.5) ng/mL, p 0.0116; IL-6, 572 (93–1000) vs 38 (17–120) pg/mL, p 0.0006].

**Table 7:** General characteristics of different infection sources in the ALI group

Characteristics	Abdomen, n = 10	Pulmonary, n = 14	p
Age, years	62.5 (54.8–78.3)	65.5 (60.8–69.8)	0.7933
Sex, n (%)			
Male	6 (60.0)	10 (71.4)	
Mortality, n (%)	3 (30.0)	1 (7.1)	
ICU stay time, days	8.5 (4.5–27.5)	20.5 (13.5–41.8)	0.1693
APACHE II	18.0 (12.3–23.3)	17.5 (12.8–26.5)	0.5804
SOFA	8.5 (6.3–10.0)	8.5 (6.3–11.0)	0.9829
mtDNA, log copies/µL	5.14 (4.39–5.87)	4.79 (2.52–5.54)	0.0492*
OI, mmHg	215 (178–243)	256 (230–271)	0.0369*
PEEP, cmH2O	7.0 (6.0–8.0)	5.0 (5.0–6.0)	0.1096
Pplat, cmH2O	19.0 (17.0–23.0)	19.0 (17.5–21.5)	0.8834
MAP, mmHg	83.5 (79.5–88.5)	85.5 (79.8–92.3)	0.1795
HR, bpm	100.0 (95.0–104.5)	84.0 (70.0–91.0)	0.0176*
CVP, mmHg	10.0 (8.5–11.0)	9.0 (7.8–10.3)	0.6752
NE, µg/kg/min	0.23 (0.06–0.73)	0.11 (0.05–0.18)	0.0427*
Lactate, mmol/L	2.0 (1.0–4.4)	1.5 (1.1–1.7)	0.0534
PCT, ng/mL	8.1 (3.2–93.3)	2.0 (0.8–6.5)	0.0116*
IL-6, pg/mL	572 (93–1000)	38 (17–120)	0.0006**
IL-8, pg/mL	198 (121–500)	108 (39–192)	0.0808
IL-10, pg/mL	11 (7–56)	6 (5–10)	0.4274
TNFα, pg/mL	25 (14–35)	19 (13–28)	0.6741

*ALI* acute lung injury, *ICU* intensive care unit, *APACHE* Acute Physiology and Chronic Health Evaluation, *SOFA* Sequential Organ Failure Assessment, *OI* oxygen index, *PEEP* positive end expiratory pressure, *Pplat* plat pressure, *MAP* mean arterial pressure, *HR* heart rate, *CVP* central venous pressure, *NE* norepinephrine, *PCT* procalcitonin, *CRP* C-reactin protein, *IL-6* interleukin 6, *IL-8* interleukin 8, *IL-10* interleukin 10, *TNFα* tumor necrosis factor α

## Discussion

To explore the relationship between circulating mtDNA and septic lung injury, we investigated the level of plasma mtDNA within 24 h after admission in different septic patient groups. We revealed that higher plasma mtDNA levels were associated with ALI incidence and mortality in septic patients. The association was stronger in patients with abdominal infection than in those with pulmonary infection.

Mounting evidence has revealed that extracellular mtDNA might be useful as a biomarker in critically ill patients and is associated with disease severity and mortality [16–18]. Circulating mtDNA may contribute to the mechanism linking distant injury to subsequent lung injury. An increasing number of studies have found elevated mtDNA levels in hip fracture patients with lung injury [19, 20], an acute respiratory distress syndrome (ARDS) population from a mixed ICU unit [6] and a cohort of trauma patients [21]. In our study, we further explored whether higher plasma mtDNA levels were associated with ALI incidence and mortality in septic patients. Plasma mtDNA levels were significantly increased in the group of septic patients with ALI compared with those in the control group patients [5.01 (3.38–6.64) vs 4.13 (3.20–5.07) log copies/µL, p 0.0172]. The sensitivity of other inflammatory biomarkers, such as procalcitonin, CRP IL-6, IL-8, IL-10 and TNFα, was inferior to mtDNA among septic.

Consistent with a previous study, we revealed that the mtDNA level (HR, 3.2052; 95% CI 1.1608–8.8500; p 0.0253) was independently associated with mortality, and the area under the ROC curve was 0.856 (95% CI 0.748–0.930). Patients with high mtDNA levels had worse outcomes, and post hoc tests showed significant differences in 28-day survival rates (log-rank [Mantel-Cox], 6.842; p 0.0089). Patients with high mtDNA levels experienced longer ICU stay times [21.5 (11.3–42.8) vs 6.0 (4.0–12.0) days, p < 0.0001], more severe organ dysfunction [SOFA score, 10.9 (7.6–14.2) vs 6.8 (3.8–9.7), p < 0.0001] and worse respiratory and hemodynamic conditions.

MtDNA level (odds ratio 2.7506; 95% CI 1.1647–6.4959; p 0.0210) was also independently associated with ALI risk. The interaction between mtDNA and innate immunity helps us to understand the mechanism of SIRS caused by injury, such as trauma or sepsis. Due to the bacterial ancestry of mitochondria, mtDNA contains unmethylated CpG repeats and formylated peptides that could be recognized by pattern-recognition receptors [22, 23]. mtDNA released into the cytosol or circulation could activate the TLR-9-mediated NF-κB pathway [5, 8] or interact with inflammasomes, leading to caspase-1 activation or secretion of proinflammatory cytokines such as IL-1β and IL-18 [3, 24]. Based on the molecular biology of mtDNA as a potent DAMP, circulating mtDNA may contribute to the mechanism linking distant injury to subsequent lung injury. Therefore, we hypothesized that the association between circulating mtDNA and septic ALI would be strongest in patients with extrapulmonary sepsis sources. The basic clinical characteristics of all patients showed that bloodstream and abdominal infection seemed to be more common than pulmonary infection in patients with ALI. As we expected, a higher amount of mtDNA [5.14 (4.39–5.87) vs 4.79 (2.52–5.54) log copies/µL, p 0.0492] was seen in patients with abdominal infection than in those with pulmonary infection. The OI of abdominal infection patients was even worse than that of pulmonary infection patients [215 (178–243) vs 256 (230–271) mmHg, p 0.0369]. Consistent with our hypothesis, higher levels of inflammatory biomarkers such as PCT and IL-6 and higher HR and NE doses all indicated a more severe inflammatory response in abdominal infection.

There are still several limitations in our study. This is a single center study, which may limit generalizability. A larger sample size or multicenter study and subgroup analysis, especially among patients with specific infectious sources, may help. Second, an increasing number of studies have suggested that mtDNA copy numbers may vary among different disease courses [21, 25]. Our study collected only blood samples within 24 h after admission, and a dynamic view of mtDNA copy number may be significant and worthy of further study.

## Conclusions

Plasma mtDNA levels within 24 h after admission were associated with ALI incidence and mortality in septic patients in our study. The association was stronger in patients with extrapulmonary infection sources than in those with pulmonary infection.

## Data Availability

The datasets supporting the conclusions of the current study are available from the corresponding author upon reasonable request. Please contact the corresponding author if you would like to request the dataset.

## Abbreviations

mtDNA: Mitochondrial DNA
ALI: Acute lung injury
RV: Right ventricular
DAMP: Damage-associated molecular pattern
TLR-9: Toll-like receptor 9
SIRS: Systemic inflammatory response syndrome
PUMCH: Peking Union Medical College Hospital
ICU: Intensive care unit
SOFA: Sequential Organ Failure Assessment
OI: Oxygen index
ASE: American Society of Echocardiography
TAPSE: Tricuspid annular plane systolic excursion
APACHE: Acute Physiology and Chronic Health Evaluation
PCR: Polymerase chain reaction
ROC: Receiver operating characteristic
Pplat: Plat pressure
PEEP: Positive end expiratory pressure
CVP: Central venous pressure
MAP: Mean arterial pressure
HR: Heart rate
NE: Norepinephrine
PCT: Procalcitonin
CRP: C-reactin protein
IL-6: Interleukin 6
IL-8: Interleukin 8
IL-10: Interleukin 10
TNFα: Tumor necrosis factor α
ARDS: Acute Respiratory Distress Syndrome
PMNs: Polymorphonuclear neutrophils
